# Method-Specific Suicide Mortality Trends in Australian Men from 1978 to 2017

**DOI:** 10.3390/ijerph18094557

**Published:** 2021-04-25

**Authors:** Noelia Lucía Martínez-Rives, Bibha Dhungel, Pilar Martin, Stuart Gilmour

**Affiliations:** 1 Department of Psychiatry and Social Psychology, University of Murcia, 30100 Murcia, Spain; noelialucia.martinezr@um.es (N.L.M.-R.); mpmartin@um.es (P.M.); 2 Graduate School of Public Health, St. Luke’s International University, Tokyo 104-0045, Japan; sgilmour@slcn.ac.jp; 3 Department of Health Policy, National Centre for Child Health and Development, Tokyo 157-0074, Japan

**Keywords:** suicide, Australia, method-specific, men, hanging, trends, mortality, suicide mortality, states, New South Wales

## Abstract

In 2017 Australia saw the highest overall suicide rate in the past 10 years, with male suicide rates three times higher than in women. Since the mid-1980s there have been major changes in suicide epidemiology in Australia with large shifts in method of suicide among both men and women. This study examined method-specific suicide trends in Australian men over the past 40 years by state. Suicide mortality data for the period 1978 to 2017 was obtained from the Australian Institute of Health and Welfare (AIHW) National Mortality Database and log-linear Poisson regression analysis was used to analyse suicide mortality. This study found large differences between states in patterns and trends in suicide mortality from 1978 to 2017. Hanging, gas and firearms were the most common methods of suicide in Australia. We found statistically significant increasing trends in hanging suicide among men in all six states. The study findings highlight the growing concern of hanging-related suicide in all states in Australia since the late 1970s. New suicide prevention strategies focusing on the ubiquity and ease of hanging as a method will be needed in order for Australia to reduce suicide mortality in future.

## 1. Introduction

The prevalence of suicide methods differs between countries. Worldwide, hanging is the predominant method for both men and women, particularly in high-income countries [1], except for the USA, where firearms are most often used as a method of suicide [2]. Poisoning, mainly with pesticides, is the most common suicide method in Asia [3] and in other countries where pesticides are easily accessible and in low- and middle-income countries [4]. Jumping from a height as a means of suicide is common in highly urbanized areas such as Singapore and Hong Kong [5,6].

In higher-income countries, the incidence of male suicide is three to four times greater than that of female suicide, due in part to the fact that men use more lethal means, such as hanging, while women use methods such as drug overdose, which is less likely to cause death, and women are also more likely to seek medical attention after intentional self-harm [7]. The relevant factors driving these global and sex-based differences in the choice and prevalence of suicide methods appear to be physical and cognitive availability, lethality and socio-cultural acceptability [8,9,10]. Understanding patterns and trends in methods is essential to developing strategies to reduce suicide, especially given that strategies based around restricting access to means of suicide can be highly effective [1,11].

Suicide, which is the 13th overall leading cause of death in Australia, constitutes 2% of overall annual deaths [12,13]. It remains a major public health problem in Australia and is listed as one of the leading causes of death among Australians aged 15 to 44 years [13]. Suicide constitutes the top cause for the highest years of potential life lost [13] and every year over 65,000 people in Australia attempt suicide [14]. Australian suicide rates have risen from 10.2 per 100,000 inhabitants in 2006 to 12.9 suicide deaths per 100,000 people in 2019 and a suicide rate of 19.8 per 100,000 among men [15].

In 2017 suicide mortality rates among Indigenous Australians were almost twice those of the non-indigenous Australian population [13], and suicide deaths in Indigenous Australians are typically younger than in non-Indigenous Australians (23 years versus 37.8 years). The incidence of suicide mortality among indigenous Australians is highest in the states of New South Wales (NSW) and Queensland [16]. Previously, some studies have identified differences in the suicide methods used by young Australian indigenous people in Queensland, observing an increase in the frequency of suicides by hanging and inhalation of motor vehicle gases and a decrease in suicides committed with firearms [17]. Past research has suggested that indigenous suicides are more likely to involve hanging-related methods, based on the relative ease of availability of ligatures in remote communities [18].

Aside from these broad assessments of recent levels of suicide mortality, few studies comprehensively examined epidemiological trends or changes in choice of suicide method in each Australian state over an extended period. Past studies have focused on comparing suicide rates in Australia with other countries, like the one of Snowdon, Saberi and Moazen-Zadeh [19], addressing a single suicide method or a state in the country, such as that of Too, Bugeja, Milner, McClure and Spittal [20], but no comprehensive assessment has been conducted of the changing patterns of suicide method over the past decades in Australia.

The objective of the current study is to examine the trends in the most used suicide method in each Australian state from 1978 to 2017. Specifically, this study will focus on suicide among men, the population with the most suicide deaths (75.4% of deaths by suicide) in adults aged over 20, in which the highest suicide rates were also found [15,16].

## 2. Materials and Methods

### 2.1. Data Source

Data on male suicide deaths from 1978 to 2017 were obtained from the Australian Institute of Health and Welfare (AIHW) National Mortality Database [21] and the Australian Bureau of Statistics [15,22]. Cause of Death Unit Record File data are provided to the AIHW by the Registries of Births, Deaths and Marriages and the National Coronial Information System (managed by the Victorian Department of Justice) and include cause of death coded by the Australian Bureau of Statistics (ABS).

The causes of death analysed in this study are classified according to the ICD-10 codes [23] as deaths derived from intentionally self-inflicted injuries. Deaths from previous years were mapped to ICD-10 codes according to Taylor [24] and ICD9-ICD10 mapping tables released by the World Health Organization (WHO). Following the WHO [23] codes, suicide rates were calculated for hanging, strangulation and suffocation (ICD-10, X70); poisoning with gas (ICD-10, X67); non-gas poisoning (ICD-10, X60–66, X68 and X69); by handgun discharge (ICD-10, X72–X75); drowning (ICD-10, X71); and other causes (ICD-10, X84), for each Australian state for each year, between 1978–2017.

As the characteristics of each Australian state could give variations in the tendencies of the suicide method, the states were analysed separately.

### 2.2. Statistical Analysis 

The 2001 census population was used to standardise the rates across all years as per the standard procedure adopted by the Australian Bureau of Statistics and the Australian Institute of Health and Welfare [25,26] to enable comparability between years, for graphing and overall comparison. Age was summarized into four categories, 20–34, 35–49, 50–64 and 65+. As children under 20 years had very low suicide rates they were not included in the analysis. Six categories of suicide methods were examined which included hanging, gun, drowning, gas, poisoning and all other methods. In the dataset, less than 3 deaths were coded as < 3 for the privacy of the deceased, which were converted to 2 for analysis. The suicide category ‘Others’ was generated by subtracting deaths due to hanging, gun, gas, drowning and poisoning from total suicide deaths as provided in the dataset. Some of the values generated were negative, possibly, as all deaths < 3 were converted to 2. Such negative values were changed to zero for analysis. A variable named NFA (National Firearms Agreement) was generated with values 0 for the years 1996 and before, and 1 for the years 1997 and after to control for any possible effect of the national firearms agreement [27].

First, a descriptive approach was applied to investigate the most notable suicide rates and the most characteristic patterns of suicide mortality by method, separately by age and/or state. Then, in order to analyse the trends in the method in each of the states throughout the selected period, a Poisson regression model was used. A two-way interaction model of the method and year was used to test for significant differences in trend by method within each state. Linear combinations of the key variables were calculated to describe the trends in suicide by method. All these statistical analyses were conducted in Stata IC version 15.1 (StataCorp LP, College Station, TX, USA).

## 3. Results

**Figure 1:**
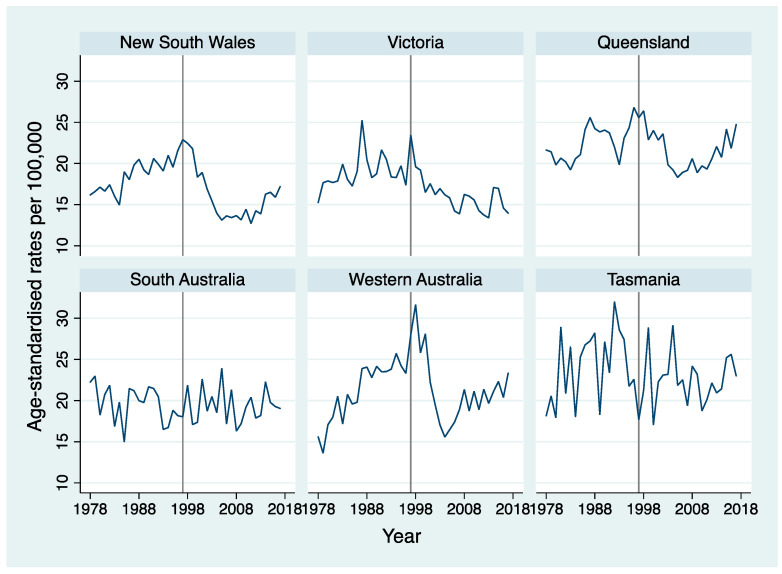
Age-standardised overall suicide mortality rate per 100,000 by state among men. The grey line in 1997 represents the year when National Firearms Agreement was introduced in Australia and separates the 40-year study period into two halves.

Figure 1 shows the overall trends in age-standardised suicide mortality rates from 1978 to 2017 separately by state. Rates of suicide started declining in New South Wales, Victoria, Queensland and Western Australia since late 1990s. In the last decade, however, suicide rates have been increasing in most of these states.

**Figure 2:**
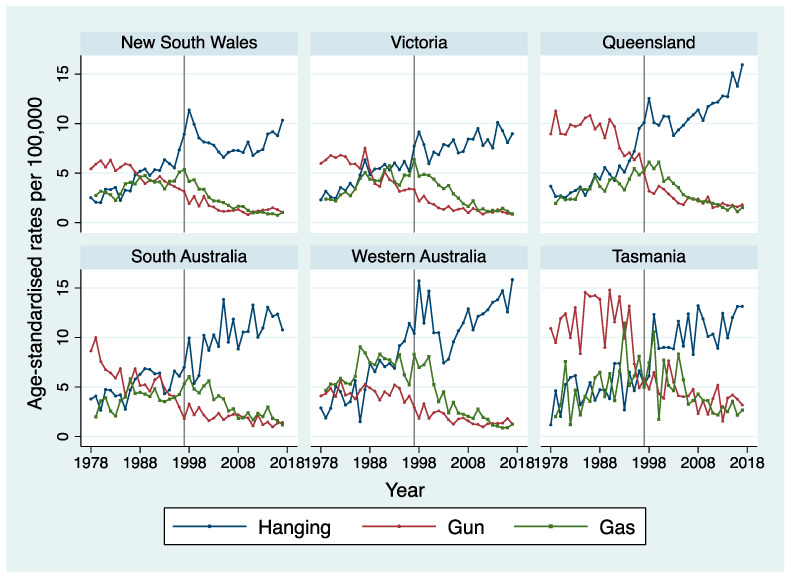
Age-standardised suicide mortality rate per 100,000 by method separately by state among men. The grey line in 1997 represents the year when the National Firearms Agreement was introduced in Australia and separates the 40-year study period into two halves.

Figure 2 shows the method-specific trends in age-standardised suicide rates from 1978 to 2017 separately by state. Hanging, gas and firearms were the most common methods of suicide in Australia. Mortality by hanging has been rising since the late 1970s, while trends in suicide by gun and gas have been declining since the beginning of the study period.

### Regression Analysis

Table 1 and Table 2 show the results of the Poisson regression models of suicide mortality by state. From these tables, it can be seen that the poisoning suicide rate has decreased significantly (incidence rate ratio (IRR) < 1) throughout the chosen time interval (1978–2017) in all states, except Tasmania (IRR = 0.999, 95% CI: 0.986–1.012), where there has been no significant trend in poisoning suicide in this state. In contrast, the interaction term for method and time shows a significantly higher time trend for hanging in every state, with IRRs of 1.043–1.054 in every state. Only hanging and ‘others’ suicide had significantly higher trends than poisoning, indicating that these are the only methods of suicide that are increasing. Suicide mortality also declined with age and hanging and firearm-related suicides were much higher than poisoning at the beginning of the study period, though long-term trends. The overall suicide mortality rate decreased in New South Wales, Western Australia and Tasmania since the mid-1990s, which coincides with the implementation of Australia’s first major national suicide prevention strategy, while the rates increased in Victoria, Queensland and South Australia. The results were, however, statistically significant only in Western Australia.

Table 3 shows the method-specific trend in suicide mortality rate separately by state. The trend in suicide mortality rates by hanging was increasing significantly (IRR 1.026–1.042) in all six states from 1978 to 2017, with the highest increase in Western Australia (IRR = 1.45, 95% CI: 1.22–1.73), followed by New South Wales (IRR = 1.24, 95% CI: 1.13–1.36) and Victoria (IRR = 1.14, 95% CI: 1.02–1.28). The trends were decreasing by over 13% for suicide by gun, gas and poisoning in all states except Tasmania, where the results were not significant. Suicide by drowning increased slightly in Western Australia (IRR = 1.007, 95% CI: 0.993–1.021), however, the results were not statistically significant.

Regarding other suicide methods (‘others’), a significant increase was also found in all states, especially in Queensland (IRR = 1.03, 95% CI: 1.02–1.03) and Tasmania (IRR = 1.03, 95% CI: 1.01–1.06).

## 4. Discussion

This study found large differences between states in patterns and trends in suicide mortality from 1978 to 2017. The main results obtained in the present study indicate that in Australia, since the end of the 1970s, hanging has been increasing as a method of suicide, finding, at the same time, a downward and statistically significant trend in the use of firearms and inhalation of gases for the same purpose [28]. This indicates that since the mid-1980s there has been an important change in the epidemiology of suicide, in terms of the method used [22,29].

Previous studies [30] have identified a peak in suicides occurring in 1997 and found a statistically significant reduction in the suicide rate in men in Australia between 1998 and 2007 [31] although one study that disaggregated methods by method identified this peak as primarily happening in hanging-related suicides [27]. In fact, when analysed over a longer time period and properly separated by method, it becomes clear that the downward trend in total suicide over the past 20 years has been driven by declines in firearm- and gas-related suicides. As suicide by these methods reaches its minimum and begins to stop declining, the continued growth of hanging suicides will likely drive a resurgence of suicide mortality. This phenomenon can already be seen in the states of Queensland, Western Australia and New South Wales and will likely become evident in other states and at the national level in the near future.

As can be seen in Figure 2, in the first half of the period covered by this study a predominant use of firearms is shown over the rest of the methods, in all states, except Western Australia. Over the period of the study, this trend declines, likely due to improvements in firearm laws since the mid-1980s and urbanisation. Past research has found high rates of suicide by firearms in Australian men in rural areas [28,32], with the possibility of limited access to suicide prevention services in these settings being associated with a greater risk of suicide deaths.

Although Australia was one of the first countries to develop a national strategic approach to suicide prevention, great difficulties have been encountered in achieving this goal, taking into consideration the acceptability, availability and lethality of the most widely used methods, such as hanging, when developing prevention plans [33]. More specifically, some states like New South Wales have a state-wide suicide prevention program [34]. In addition, there have been numerous attempts to adopt measures to control suicide (by reducing access to certain drugs, reducing the toxicity of the gases expelled by cars and some household chemicals, and restricting access to firearms) [35]. However, over time it appears that these suicide prevention policies have not been effective in controlling the growth of hanging-related suicide, which is very difficult to prevent due to the ease of access to methods and its lethality.

To reduce hanging-related suicides, it is necessary to enhance early interventions that provide resources to the individual in the face of suicidal crises, beyond limiting the availability of means. Without further enhanced suicide prevention strategies targeting ideation, mental health crises, and support networks, the gains of the past 20 years will begin to be undone.

## 5. Conclusions

The study findings highlight the growing concern of hanging-related suicide in all states in Australia since the late 1970s. This change in the balance of suicide methods is likely the primary driver of the recent increase in the total suicide rate among men and will continue to drive upward trends in suicide rates so long as interventions are not developed targeting this method specifically. While the suicide prevention strategies of the past 20 years appear to have been very effective, alongside gun control measures, in preventing other forms of suicide, a new generation of strategies is needed if Australia is to maintain its success in reducing suicide mortality in the future.

This study has limitations in that the results of the analysis by age groups show the percentage of men who commit suicide by state, but it does not provide data on the injurious method predominantly used specifically based on race or ethnicity, but rather generally. Another aspect that could be a limitation is the inability of the present work to shed light on female suicides, having focused on male suicide trends only. Male suicides need to be examined separately to female suicides, however, because patterns of suicide method and risk factors differ significantly between the genders. In future, a follow-up study in women could be considered, since in another recent study [29] an increasing trend was observed in the number of suicides by hanging also among young women (10–14 years) between 2004 and 2014, although specific factors that might be associated with this rise were not examined. However, this study has several strengths that we can highlight, such as the use of the Poisson regression model for data analysis, which meets the equi-dispersion criterion, observing heterogeneity in the data depending on the method. It is also pertinent to highlight the relevance of analysing the changes that have occurred in the mechanisms through which suicide is carried out in the different states of the country and over a long period, thus enabling specific patterns to be disaggregated by state, which is the administrative level at which health interventions are usually managed in Australia [4]. Additionally, this study provides information on suicides in the Australian population in four age groups (20–34, 35–49, 50–64 and over 65 years), selecting from all the available data, those who are most affected by this phenomenon.

In view of the findings in this study, in future research, it is necessary to examine other more population-specific social or psychological variables that may be related to trends in suicide method. Our research in particular shows the importance of understanding drivers of the choice of hanging as a suicide method, and the need for research on new strategies to prevent this form of suicide.

Although the results of this study cannot be generalized to other countries, it can serve as a reference for those in a similar situation with regard to their suicide prevention strategies. The National Suicide Prevention Implementation Strategy 2020–2025: Working together to save lives [36] has recently been published in Australia. Although past strategies appear to have been successful in driving down many causes of suicide, our study has identified the need for a new generation of preventive methods specifically targeting hanging-related suicides, if Australia is not to see future large increases in suicide mortality across many states.

## Figures and Tables

**Table 1:** Poisson regression analysis of suicide mortality in New South Wales, Victoria, and Western Australia.

Variables	Incidence Rate Ratio	95% Confidence Interval
	New South Wales	
Year	0.984	(0.980–0.988) *
Suicide Method		
Hanging	1.241	(1.130–1.363) *
Gun	2.367	(2.153–2.603) *
Drowning	0.167	(0.137–0.204) *
Gas	1.731	(1.570–1.909) *
Poisoning	Reference	
Others	0.785	(0.704–0.875) *
Age Category		
20–34 years	Reference	
35–49 years	0.982	(0.949–1.017)
50–64 years	0.850	(0.818–0.884) *
65+ years	0.973	(0.933–1.015)
Period		
Before national firearms agreement		
After national firearms agreement	0.971	(0.918–1.027)
Year-Method interaction		
Year-Hanging	1.043	(1.039–1.047) *
Year-Gun	0.967	(0.963–0.972) *
Year-Drowning	1.001	(0.992–1.010)
Year-Gas	0.986	(0.981–0.990) *
Year-Poisoning	Reference	
Year-Others	1.020	(1.016–1.025) *
	Victoria	
Year	0.975	(0.970–0.980) *
Suicide Method		
Hanging	1.147	(1.028–1.280) *
Gun	2.595	(2.327–2.895) *
Drowning	0.232	(0.189–0.286) *
Gas	1.627	(1.453–1.821) *
Poisoning	Reference	
Others	0.714	(0.628–0.812) *
Age Category		
20–34 years	Reference	
35–49 years	0.983	(0.945–1.023)
50–64 years	0.873	(0.835–0.913) *
65+ years	0.894	(0.850–0.939) *
Period		
Before national firearms agreement	Reference	
After national firearms agreement	1.020	(0.956–1.088)
Year-Method interaction		
Year-Hanging	1.052	(1.047–1.057) *
Year-Gun	0.967	(0.962–0.972) *
Year-Drowning	0.997	(0.987–1.006)
Year-Gas	1.001	(0.995–1.006)
Year-Poisoning	Reference	
Year-Others	1.026	(1.020–1.031)
	Western Australia	
Year	0.987	(0.980–0.994) *
Suicide Method		
Hanging	1.460	(1.226–1.738) *
Gun	1.922	(1.593–2.319) *
Drowning	0.145	(0.100–0.212)
Gas	3.123	(2.626–3.713) *
Poisoning	Reference	
Others	0.412	(0.323–0.526) *
Age Category		
20–34 years	Reference	
35–49 years	0.914	(0.864–0.967) *
50–64 years	0.684	(0.639–0.731) *
65+ years	0.674	(0.624–0.728) *
Period		
Before national firearms agreement	Reference	
After national firearms agreement	0.999	(0.904–1.105)
Year-Method interaction		
Year-Hanging	1.045	(1.038–1.053) *
Year-Gun	0.975	(0.967–0.983) *
Year-Drowning	1.020	(1.005–1.035) *
Year-Gas	0.974	(0.966–0.981) *
Year-Poisoning	Reference	
Year-Others	1.029	(1.020–1.039) *

* Significant at 5% level of significance.

**Table 2:** Poisson regression analysis of suicide mortality in Queensland, South Australia and Tasmania.

Variables	Incidence Rate Ratio	95% Confidence Interval
	Queensland	
Year	0.986	(0.981–0.991) *
Suicide Method		
Hanging	0.960	(0.847–1.088)
Gun	3.827	(3.403–4.303) *
Drowning	0.212	(0.166–0.271) *
Gas	1.337	(1.170–1.527) *
Poisoning	Reference	
Others	0.383	(0.323–0.453) *
Age Category		
20–34 years	Reference	
35–49 years	1.010	(0.970–1.052)
50–64 years	0.808	(0.771–0.847) *
65+ years	0.851	(0.808–0.897) *
Period		
Before national firearms agreement	Reference	
After national firearms agreement	1.009	(0.943–1.080)
Year-Method interaction		
Year-Hanging	1.054	(1.049–1.059) *
Year-Gun	0.960	(0.955–0.965) *
Year-Drowning	0.992	(0.981–1.002)
Year-Gas	0.996	(0.991–1.002)
Year-Poisoning	Reference	
Year-Others	1.032	(1.025–1.038) *
	South Australia	
Year	0.980	(0.973–0.988) *
Suicide Method		
Hanging	1.050	(0.881–1.251)
Gun	2.354	(1.974–2.809) *
Drowning	0.264	(0.192–0.364) *
Gas	1.335	(1.110–1.607) *
Poisoning	Reference	
Others	0.322	(0.249–0.416) *
Age Category		
20–34 years	Reference	
35–49 years	0.966	(0.905–1.031)
50–64 years	0.854	(0.793–0.920) *
65+ years	0.935	(0.862–1.014)
Period		
Before national firearms agreement	Reference	
After national firearms agreement	1.127	(1.017–1.249) *
Year-Method interaction		
Year-Hanging	1.051	(1.043–1.058) *
Year-Gun	0.967	(0.958–0.975) *
Year-Drowning	0.989	(0.974–1.004)
Year-Gas	1.002	(0.994–1.011)
Year-Poisoning	Reference	
Year-Others	1.028	(1.017–1.039) *
	Tasmania	
Year	0.999	(0.986–1.012)
Suicide Method		
Hanging	1.095	(0.795–1.509)
Gun	4.779	(3.582–6.377) *
Drowning	0.487	(0.310–0.768) *
Gas	1.788	(1.300–2.458) *
Poisoning	Reference	
Others	0.143	(0.078–0.261) *
Age Category		
20–34 years	Reference	
35–49 years	0.900	(0.807–1.004)
50–64 years	0.843	(0.749–0.948) *
65+ years	0.883	(0.777–1.005)
Period		
Before national firearms agreement	Reference	
After national firearms agreement	0.849	(0.713–1.009)
Year-Method interaction		
Year-Hanging	1.043	(1.029–1.057) *
Year-Gun	0.969	(0.956–0.982) *
Year-Drowning	0.994	(0.974–1.015)
Year-Gas	1.000	(0.986–1.014)
Year-Poisoning	Reference	
Year-Others	1.040	(1.016–1.064) *

* Significant at 5% level of significance.

**Table 3:** Change in mortality trend by suicide method separately by all six states.

Suicide method	Incidence Rate Ratio	95% Confidence Interval
	**New South Wales**	
Hanging	1.026	(1.023–1.029) *
Gun	0.952	(0.948–0.955) *
Drowning	0.985	(0.976–0.993) *
Gas	0.970	(0.966–0.973) *
Poisoning	0.984	(0.980–0.988) *
Others	1.004	(1.000–1.007)
	**Victoria**	
Hanging	1.026	(1.022–1.029) *
Gun	0.943	(0.939–0.947) *
Drowning	0.972	(0.963–0.981) *
Gas	0.976	(0.972–0.980) *
Poisoning	0.975	(0.970–0.980) *
Others	1.000	(0.996–1.005)
	**Queensland**	
Hanging	1.039	(1.036–1.043) *
Gun	0.946	(0.942–0.950) *
Drowning	0.978	(0.968–0.988) *
Gas	0.982	(0.978–0.987) *
Poisoning	0.986	(0.981–0.991) *
Others	1.017	(1.011–1.023) *
	**South Australia**	
Hanging	1.030	(1.024–1.035) *
Gun	0.948	(0.941–0.954) *
Drowning	0.969	(0.955–0.984) *
Gas	0.982	(0.976–0.989) *
Poisoning	0.980	(0.973–0.988) *
Others	1.008	(0.998–1.017)
	**Western Australia**	
Hanging	1.032	(1.027–1.037) *
Gun	0.963	(0.956–0.969) *
Drowning	1.007	(0.993–1.021)
Gas	0.961	(0.956–0.967) *
Poisoning	0.987	(0.980–0.994) *
Others	1.017	(1.008–1.025) *
	**Tasmania**	
Hanging	1.042	(1.032–1.052) *
Gun	0.968	(0.958–0.977) *
Drowning	0.993	(0.975–1.011)
Gas	0.999	(0.989–1.010)
Poisoning	0.999	(0.986–1.012)
Others	1.039	(1.017–1.061) *

* Significant at 5% level of significance.

## Data Availability

Not applicable.

## References

[1] Dhungel B., Sugai M.K., Gilmour S. (2019). Trends in suicide mortality by method from 1979 to 2016 in Japan. Int. J. Environ. Res. Public Health.

[2] Ajdacic-Gross V., Weiss M.G., Ring M., Hepp U., Bopp M., Gutzwiller F., Rössler W. (2008). Methods of suicide: International suicide patterns derived from the WHO mortality database. Bull. World Health Organ..

[3] Wu K.C.-C., Chen Y.-Y., Yip P.S.F. (2012). Suicide methods in Asia: Implications in suicide prevention. Int. J. Environ. Res. Public Health.

[4] World Health Organization (2014). Preventing Suicide: A Global Imperative.

[5] Chia B.-H., Chia A., Ng W.-Y., Tai B.-C. (2011). Suicide Methods in Singapore (2000–2004): Types and Associations. Suicide Life Threat. Behav..

[6] Wong P.W.C., Caine E.D., Lee C.K.M., Beautrais A., Yip P.S.F. (2013). Suicides by jumping from a height in Hong Kong: A review of coroner court files. Soc. Psychiatry Psychiatr. Epidemiol..

[7] Milner A., King T. (2018). Men’s work, women’s work and suicide: A retrospective mortality study in Australia. Aust. N. Z. J. Public Health.

[8] Barber C.W., Miller M.J. (2014). Reducing a suicidal person’s access to lethal means of suicide: A research agenda. Am. J. Prev. Med..

[9] Florentine J.B., Crane C. (2010). Suicide prevention by limiting access to methods: A review of theory and practice. Soc. Sci. Med..

[10] Kõlves K., de Leo D. (2016). Suicide methods in children and adolescents. Eur. Child Adolesc. Psychiatry.

[11] Hawton K. (2007). Restricting access to methods of suicide: Rationale and evaluation of this approach to suicide prevention. Crisis.

[12] Australian Institute of Health and Welfare Deaths in Australia, Leading Causes of Death, Table S3.1. https://www.aihw.gov.au/reports/life-expectancy-death/deaths-in-australia/contents/leading-causes-of-death.

[13] Australian Bureau of Statistics Causes of Death. https://www.abs.gov.au/statistics/health/causes-death/causes-death-australia/2019#intentional-self-harm-suicides-key-characteristics.

[14] Slade T., Johnston A., Teesson M., Whiteford H., Burgess P., Pirkis J., Saw S. (2009). The Mental Health of Australians 2: Report on the 2007 National Survey of Mental Health and Wellbeing.

[15] Australian Bureau of Statistics Cause of Death, Australia, 2016–Intentional Self-Harm: Key Characteristics. https://www.abs.gov.au/ausstats/abs@.nsf/Lookup/by%20Subject/3303.0~2016~Main%20Features~Intentional%20self-harm:%20Key%20characteristics~7.

[16] Australian Bureau of Statistics Estimates of Aboriginal and Torres Strait Islander Australians. https://www.abs.gov.au/statistics/people/aboriginal-and-torres-strait-islander-peoples/estimates-aboriginal-and-torres-strait-islander-australians/latest-release.

[17] De Leo D., Sveticic J., Milner A., Mckay K. (2011). Suicide in Indigenous Populations of Queensland.

[18] Cantor C.H., Baume P.J.M. (1998). Access to methods of suicide: What impact?. Aust. N. Z. J. Psychiatry.

[19] Snowdon J., Mehdi Saberi S., Moazen-Zadeh E. (2020). A comparison between the age patterns and rates of suicide in the Islamic Republic of Iran and Australia. East. Mediterr. Health J..

[20] Too L.S., Bugeja L., Milner A., McClure R., Spittal M.J. (2017). Predictors of using trains as a suicide method: Findings from Victoria, Australia. Psychiatry Res..

[21] Australian Institute of Health and Welfare National Mortality Database (NMD). https://www.aihw.gov.au/about-our-data/our-data-collections/national-mortality-database.

[22] Australia Bureau of Statistics Suicides Australia 1921 to 1998: ABS Finds Suicide Highest in 25–44 Age Group. https://www.abs.gov.au/AUSSTATS/abs@.nsf/mediareleasesbyCatalogue/7915C544D6345E12CA257A440014D2EC?OpenDocument.

[23] World Health Organization (2011). Appendix A: ICD-10 codes for intentional self-harm. Preventing Suicide: A Resource for Suicide Case Registration.

[24] Taylor F. (1992). Australian Institute of Health and Welfare Guide to use of international classification of diseases in Australia/Fred Taylor.

[25] Australian Bureau of Statistics Australian Demographic Statistics, Mar 2013. https://www.abs.gov.au/ausstats/abs@.nsf/Lookup/3101.0Feature+Article1Mar2013.

[26] Australian Institute of Health and Welfare (2011). Principles on the Use of Direct Age-Standardisation in Administrative Data Collections: For Measuring the Gap between Indigenous and Non-Indigenous Australians.

[27] Gilmour S., Wattanakamolkul K., Sugai M.K. (2018). The Effect of the Australian National Firearms Agreement on Suicide and Homicide Mortality, 1978–2015. Am. J. Public Health.

[28] Graham A., Reser J., Scuderi C., Zubrick S., Smith M., Turley B. (2000). Suicide: An Australian psychological society discussion paper. Aust. Psychol..

[29] Stefanac N., Hetrick S., Hulbert C., Spittal M.J., Witt K., Robinson J. (2019). Are young female suicides increasing? A comparison of sex-specific rates and characteristics of youth suicides in Australia over 2004–2014. BMC Public Health.

[30] Large M.M., Nielssen O.B., Lackersteen S.M. (2009). The rise and fall of suicide in New South Wales. Med. J. Aust..

[31] Large M.M., Nielssen O.B. (2010). Suicide in Australia: Meta-analysis of rates and methods of suicide between 1988 and 2007. Med. J. Aust..

[32] Barry R., Rehm J., de Oliveira C., Gozdyra P., Kurdyak P. (2020). Rurality and Risk of Suicide Attempts and Death by Suicide among People Living in Four English-speaking High-income Countries: A Systematic Review and Meta-analysis. Can. J. Psychiatry..

[33] Robinson J., Bailey E., Browne V., Cox G., Hooper C. (2016). Raising the Bar for Youth Suicide Prevention.

[34] Roth L., Suicide prevention (2017). NSW Parliam. Res. Serv..

[35] Yip P.S.F., Caine E., Yousuf S., Chang S.-S., Wu K.C.-C., Chen Y.-Y. (2012). Means restriction for suicide prevention. Lancet.

[36] National Suicide Prevention Project Reference Group National suicide prevention implementation strategy 2020–2025: Working together to save lives. Consultation Document to Inform the Drafting of the Strategy.

